# A Microdialysis in Adjuvant Arthritic Rats for Pharmacokinetics–Pharmacodynamics Modeling Study of Geniposide with Determination of Drug Concentration and Efficacy Levels in Dialysate

**DOI:** 10.3390/molecules23050987

**Published:** 2018-04-24

**Authors:** Ran Deng, Wei Wang, Hong Wu, Yunjing Zhang, Wenyu Wang, Li Dai, Zhengrong Zhang, Jun Fu, Feng Li

**Affiliations:** 1 College of Pharmacy, Anhui University of Chinese Medicine, Key Laboratory of Modernized Chinese Medicine in Anhui Province, Hefei 230012, China; wuhongprof@aliyun.com (R.D.); 18605538039@163.com (W.W.); daili6daisy@163.com (L.D.); zhangzhengrong311@126.com (Z.Z.); fujun6866@163.com (J.F.); fengli92@aliyun.com (F.L.); 2 Bozhou Chuangxin Technology Consulting Co. Ltd., Bozhou 236800, China; ahmxs@163.com

**Keywords:** rheumatoid arthritis, microdialysis, geniposide, PK–PD modeling, prostaglandin E_2_, UHPLC-MS/MS

## Abstract

Microdialysis, a sampling method for pharmacokinetics–pharmacodynamics (PK–PD) modeling in preclinical and clinical studies, is a convenient in vivo sampling technique. Geniposide (GE), an iridoid glycoside compound, is the major active ingredient of *Gardenia jasminoides* Ellis fruit which has an anti-inflammatory effect. In this study, an articular cavity microdialysis sampling system for adjuvant arthritic (AA) rats was established to study the effect of GE on the release of prostaglandin E_2_ (PGE_2_) in AA rats induced by Freund’s complete adjuvant (FCA). An UHPLC-MS/MS method was developed to determine the concentrations of GE and PGE_2_ in the dialysate. Through the determination of drug concentrations and PGE_2_ efficacy levels in the dialysate, the developed methods were successfully applied to set up concentration–time and effect–time profiles followed by PK–PD modeling of GE’s effect on decreasing PGE_2_ release after oral administration of GE. The effect was well described by the developed PK–PD modeling, indicating that GE may play an anti-inflammatory role via decreasing AA-induced elevated PGE_2_ levels. In the selection of suitable endogenous small molecules as effect markers, the establishment of AA rat joint-cavity microdialysis is an attractive technique for rational PK–PD studies.

## 1. Introduction

Rheumatoid arthritis (RA) is a systemic, autoimmune, acute-on-chronic inflammatory disorder that is characterized by synovial tissue hyperplasia, the formation of synovial pannus, and progressive joint damage of cartilage and bone [1,2,3]. With a global distribution, this disease, affecting about 0.5%–1.0% of adults worldwide, can result in systemic complications, progressive disability, socioeconomic costs and even early death [4,5,6]. Prostaglandin E_2_ (PGE_2_) is a metabolite of arachidonic acid produced by a COX-2-catalyzed reaction and is also a key regulatory factor derived from inflammatory cells [7]. Research has proved that the production of RA is related to the responses of organism defense, especially the inflammatory cytokines in cells such as PGE_2_. The higher the concentration of PGE_2_ in cells is, the more severe the inflammation is [8,9]. Therefore, PGE_2_ is a key inflammatory factor in the pathogenesis of RA and plays a non-negligible role in the development of RA.

Geniposide (GE) is a water-soluble iridoid glycoside that is a promising anti-inflammatory drug extracted from *Rubiaceae Gardenia* Ellis in the dry and ripe fruit [10,11]. Studies have shown that GE has anti-inflammatory, anti-oxidation and other pharmacological effects [12,13,14]. GE can alleviate the symptoms of adjuvant arthritic (AA) rats by decreasing inflammatory cytokines’ activity, increasing the levels of anti-inflammatory cytokines and decreasing the contents of inflammatory mediators [15,16,17]. In recent years, the pharmacological mechanisms of GE have been well studied; however, no studies have been available concerning its dose–effect relationship until now. Mechanisms of traditional Chinese medicine actions are so complex that it is essential for researchers to understand drug actions from different perspectives, which will be helpful to investigate these mechanisms more exhaustively and comprehensively. In this experiment, the dose of GE we chose was based on previous experimental results. Previous experiments have demonstrated through pharmacodynamics (PD) evaluation that the optimal dose of GE for oral administration to AA rats is 60 mg/kg. Therefore, in this experiment, we chose a dose lower or higher than the optimal dose, as well as the optimal dose, to observe its therapeutic effect on AA rats.

Microdialysis is a powerful in vivo sampling technique for measuring unbound drug concentrations or endogenous substances in the extracellular fluids of almost tissues (blood, brain, skin, etc.) in the body [18]. Coupled with highly selective and sensitive UHPLC-MS/MS, microdialysis is able to overcome several limitations of conventional pharmacokinetics (PK) techniques. The technology is time-resolved and space-resolved, providing free small-molecule compounds. Microdialysis for use in PK and PD research has been applied and developed, and a large amount of literature has been reported [19,20,21,22].

PK and PD are two closely related kinetic processes that are synchronized with time. PK mainly studies the relationship between the dose of a drug in vivo and time. PD mainly studies the relationship between the dose of efficacy and concentration. PK studies cannot reflect the efficacy–time relationship. Similarly, the study of PD cannot explain the relationship between concentration and time. Therefore, combining PK and PD to carry out synchronized research is necessary. The PK–PD model, a mechanism-based model, studies the time and interaction of PK–PD relationships to understand drug effects and find the optimal drug dosing regimen, which has been widely used in pre-clinical and clinical studies of drugs. In recent years, mechanism-based models have made rapid progress by insights gained into how drugs work in specific processes to improve the nature of inference and prediction [23,24].

RA is a common disease that is puzzling in the field of human health. How to develop an effective therapeutic drug for the treatment of RA and how to promote it all over the world also remain problems in the field of traditional Chinese medicine. The purposes of this study were to establish the PK and PD profiles of the joint-cavity microdialysis system in AA rats, and then to establish a PK–PD model of GE in Freund’s complete adjuvant (FCA)-induced AA rats. For PK–PD modeling, it is generally critical to seek a suitable effect biomarker that quantitatively relates to the dose, causally links to clinical outcomes, and, most importantly, can be simply sampled. Modern pharmacology studies have shown that GE, an effective component extracted from *Gardenia jasminoides*, can reduce the secretion of inflammatory cytokines in fibroblast-like synoviocytes, up-regulate the contents of anti-inflammatory cytokines, and restore the balance of proinflammatory and anti-inflammatory cytokines, thus alleviating the condition of RA. Therefore, PGE_2_, as an inflammatory cytokine, may be a useful biomarker that reflects the effects of drugs on AA rats. Therefore, in this study, selecting PGE_2_ as an effect marker and the main effective component of *Gardenia jasminoides*, GE, as the PK marker, we aimed to investigate the dose–effect relationships of GE for decreasing PGE_2_ release in AA rats using a microdialysis system.

## 2. Results 

### 2.1. Establishment of PK Study of GE in Articular Cavities of AA Rats

The UHPLC-MS/MS method for evaluating the concentration of GE in articular cavities of AA rats was validated. Pae was used as the internal standard. The regression equation of GE was *Y* = 0.00246 *X* + 1.84 × 10^−4^ (R = 0.99945). The abscissa (*X*) is the ratio of GE to the internal standard concentration, and the ordinate (*Y*) is the ratio of the measured GE peak area to the internal standard peak area. The correlation coefficient of the calibration curves for GE was >0.995. The calibration curves were linear over a concentration range of 5–4000 ng/mL for GE. The matrix effects (MEs) and recovery were evaluated for method’s validation. The MEs of different concentrations of GE (5, 250 and 4000 ng/mL) and Pae were 96.95%, 92.49%, 94.43% and 113.04%. The intraday and interday variability, accuracy (bias, %), and the relative standard deviation (RSD) were within 15%. These results showed that the UHPLC-MS/MS method provided excellent quantitative analysis of GE in microdialysate samples. Finally, the mobile phase consisting of acetonitrile (solvent A) and 0.1% formic acid in water (solvent B) was used in the experiment. The retention times were about 3.5 min for GE and about 4.05 min for Pae. The results showed that there was no interference in the peak position, and the method had a good specificity.

### 2.2. Establishment of PD Index Detection Method of PGE_2_ in Articular Cavities of AA Rats

A method of UHPLC-MS/MS for the determination of PGE_2_ in an articular dialysis solution of AA rats was established. Dexamethasone (DXM) was chosen as the internal standard. The results showed that the concentrations of PGE_2_ in the range of 100–10,000 pg/mL showed a good linear relationship with the peak area, and the regression equation was *Y* = 2.74 × 10^−5^ *X* − 0.00382 (R = 0.99992). The average recovery was 90.4%. The ratio (*X*) of PGE_2_ to the internal standard concentration is the abscissa, and the ratio (*Y*) of the peak area of PGE_2_ to the internal standard peak area is the ordinate. The precision (RSD, %) of the three concentrations (100, 2500 and 10,000 pg/mL) of GE were 5.9%, 1.1% and 0.6%, and the instrumental precision was quite appropriate. The PGE_2_ dialysis samples and internal standard were kept in good stability for 10 h without MEs at room temperature.

### 2.3. In Vitro and In Vivo Probe Recovery

**Figure 1:**
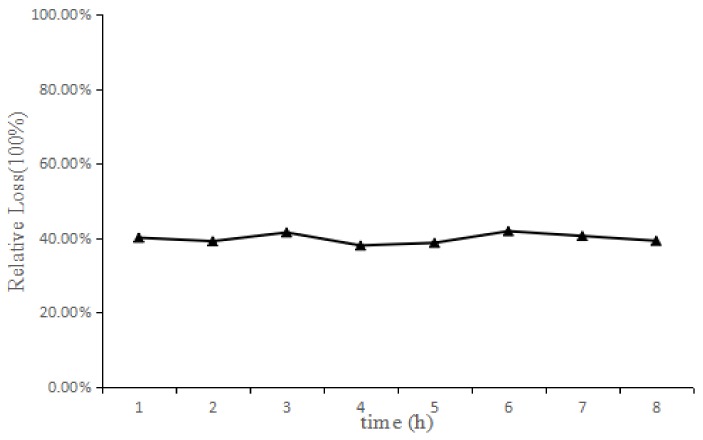
The stability of RL of knee-joint microdialysis probes in vivo (*n* = 4, $\bar{x}$ ± *s*). (The probe was perfused with Ringer containing GE (100 μg/mL) at a flow rate of 1 μL/min. The analytic samples were collected every l h to investigate the stability of the microdialysis probe in vivo over 8 h. Calculations for the joint microdialysis probe were made according to the reduction method formula: R _in vivo_ = (C_perfusate_ − C_dialysis_)/C_perfusate_ × 100%).

The effect of the flow rate and concentration on the recovery of the microdialysis probe in vivo and in vitro and the stability of the recovery rate in vivo were investigated. The results in Table 1 show that with the increase in the flow rate, the recovery rate of the joint probe decreased. The recoveries obtained by the incremental method were similar to those obtained by the reduction method, which provided the basis for the in vivo recovery of the reverse dialysis method. The results in Table 2 show that the concentrations of GE around the probe had little effect on the recovery rate. The in vitro recovery of the probe was 28.05%. The in vitro release rate of the probe was 29.07%. The changes in GE over a certain range of concentrations had no effect on the recovery rate of the probe. The in vivo recovery rate of the probe was stable over 8 h, as is shown in Figure 1. The flow rate was 1 μL/min, the sampling interval was 60 min, and the sample size was 60 μL/sample. The results of in vivo and in vitro recovery assessments indicated that the probe was stable and its recovery was independent of the analyte concentration in the joint cavity, which were suitable for this PK study.

### 2.4. PK of GE in AA Rat Joint Cavities

**Figure 2:**
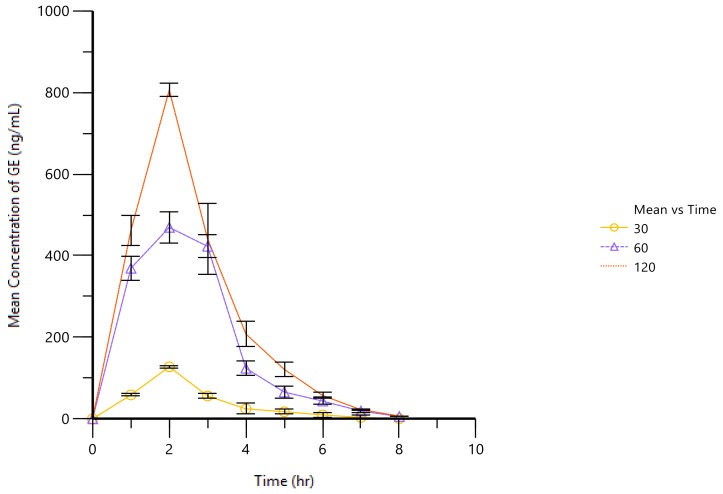
The concentration–time curve of GE after oral administration. The AA rats were treated with different concentrations of GE (30, 60 and 120 mg/kg) for 7 days. Samples were collected at the same time as the administration and once every 1 h for a total of 8 h. Values are presented as means ± SD (*n* = 6).

After the oral administration of different concentrations of GE (30, 60 and 120 mg/kg), the drug concentrations were detected in the joint cavities’ microdialysates of the AA rats. Figure 2 shows the time course of the concentrations of GE. It was successful to describe the concentrations of GE by the developed PK model. At the beginning, the concentrations of GE showed a rapid rise, reaching the maximum value after about 2 h, and then decreasing. Compared with the traditional PK method, the developed PK model can better reflect the time course of the drug and provide the experimental basis for further research into the mechanisms of GE. A one-compartment model with linear systemic elimination was able to describe the dose distribution of GE. PK parameters are listed in Table 3. It can be seen from Table 3 that GE reached the maximum concentration 2 h after oral administration. In all groups, the 60 mg/kg dose group had the highest half-life and the slowest elimination rate. The distribution volume of different doses of GE (30, 60 and 120 mg/kg) were 96.94, 42.36 and 55.46 L/kg. These results may indicate that microdialysis sampling for PK studies can provide more accurate PK data, especially data on the slow elimination of components, because microdialysis can allow continuous and long sampling without losing blood and can be used for PK studies of long-term effective compounds.

### 2.5. PGE_2_ Concentrations in Microdialysate in Articular Cavities of AA Rats

**Figure 3:**
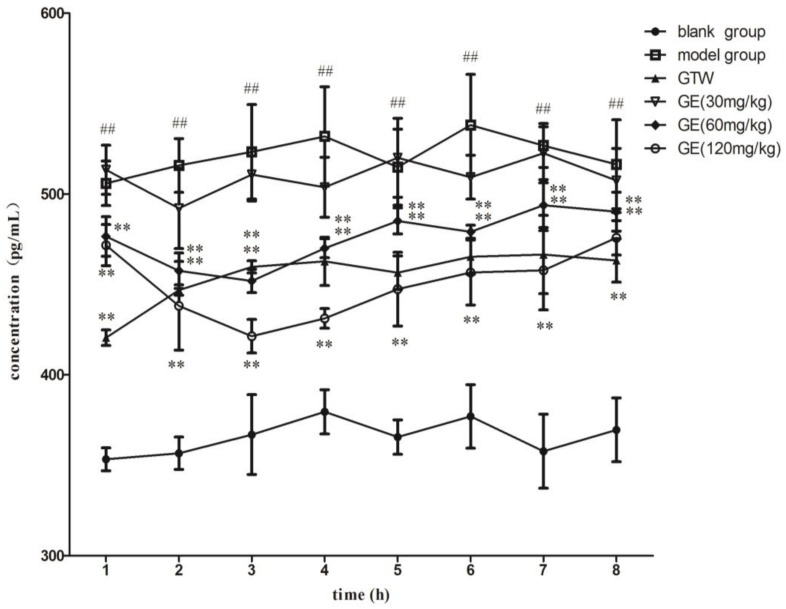
The concentration–time curve of PGE_2_. The AA rats were treated with different concentrations of GE (30, 60 and 120 mg/kg) for 7 days. Samples were collected at the same time as the administration and once every 1 h for a total of 8 h. Values are presented as means ± SD (*n* = 6); **^##^** *p* < 0.05 vs blank group; ** *p* < 0.05 vs model group.

The PGE_2_ levels of each group were measured over 8 h, as shown in Figure 3. The results showed that, compared with the blank group, the concentrations of PGE_2_ in the model group were significantly higher and remained unchanged at 8 h after implanting the probe, which indicated that the AA model was successfully established. There existed significant differences in the GE middle- and high-dose groups and *Tripterygium wilfordii* Hook F (GTW) group, compared with the model group (*p* < 0.01). Compared with the model group (*p* > 0.05), the results showed no distinct difference in the GE low-dose group. It was suggested that the middle- and high-dose groups of GE and GTW could reduce the pro-inflammatory cytokine PGE_2_ expression and regulate the relevant inflammatory cells to secrete this cytokine, thereby reducing the inflammatory reaction of AA rats so that joint damage gradually reduced.

It can be seen from Figure 2 and Figure 3 that after administration, the concentrations of PGE_2_ decreased with the increase in GE concentrations, but with the decrease in GE in vivo, the concentrations of PGE_2_ increased slightly. The results showed that the concentrations of PGE_2_ were inversely proportional to the concentrations of GE, which meant that GE had an inhibitory effect on PGE_2._ It also suggested that GE had an inhibitory effect on inflammatory markers, which may be one of the mechanisms of GE against RA. In this study, using PGE_2_ in the dialysate as an effect marker based on blood microdialysis, we found that the PD results of GE were similar to those in our previous study—both reduced the secretion of inflammatory cytokines. Because of the small interference of endogenous macromolecules, the determination of PGE_2_ levels in the dialysate is simpler and more feasible. In addition, microdialysis sampling can provide continuous and long-term effect information. Therefore, the joint-cavity microdialysis system established in this study is very reliable and suitable for the PK–PD study of endogenous small molecules as effectors.

### 2.6. PK–PD Modeling

**Figure 4:**
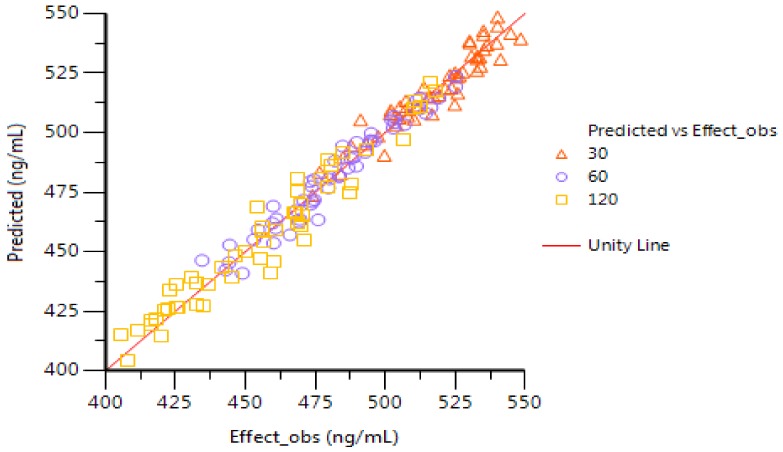
PD prediction and actual value of PGE_2_ concentrations in AA rats. Predicted values are shown by solid lines; observations are shown by hollow triangles (GE, 30 mg/kg), open circles (GE, 60 mg/kg) and hollow quadrilaterals (GE, 120 mg/kg).

Changes in PGE_2_ levels were correctly captured after AA rats were treated with different doses of GE (30, 60 and 120 mg/kg). The results of the fitting are shown in Figure 4. According to the PK–PD model, GE’s predicted reduction in PGE_2_ release in AA rats was consistent with observations, and the established model can be used to predict GE effects on the basis of dose (concentration)–effect relationships. As a time delay between the increase in GE concentration and the decrease in PGE_2_ concentration was observed, PK–PD modeling with a separated effect compartment was used for the analysis of the data. Table 4 shows PK–PD parameter estimation for the treatment effect of GE using the E_max_ model.

**Figure 5:**
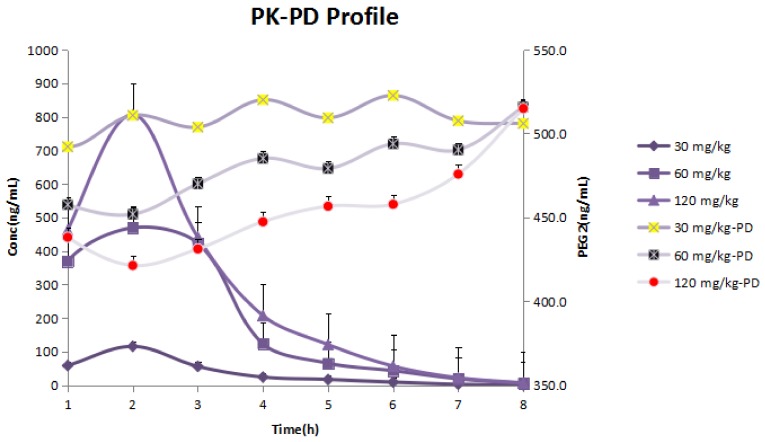
PK–PD profiles of GE in AA rats’ joint cavities. The time, PK and PD data were input into Phoenix WinNonlin 6.4, and the parameters of the PK–PD binding model were fitted. Each point shows the mean ± SD of six rats.

According to Figure 5, GE in the joint cavities reached its peak at 2 h after the oral administration of the low-, medium- and high-dose groups. The maximum inhibitory effect of PGE_2_ was observed after about 3 h in the medium- and high-dose groups. There was a lag effect between the drug effect and drug concentration, which indicated that the PK–PD joint model of GE was an indirect response model.

## 3. Discussion

RA is a common chronic, systemic and inflammatory disease. As this disease progresses, it eventually leads to severe cartilage injury and joint destruction [25,26,27]. So far, the exact pathogenesis of RA has not yet been completely confirmed. However, as it is considered a complex disease, its pathogenesis may be related to a variety of factors, including infection, hormonal disorders, genetic sensitivity and environmental factors [28,29,30].

The common animal models of experimental RA are the AA model, the collagen-induced arthritis (CIA) model, and the proteoglycan-induced arthritis (PGIA) model. Among these, AA rats have both humoral and cellular immune changes as well as many histological features in common with humans with RA. The pathological process and laboratory indexes of AA rats are similar to those of human RA. The AA model is an early and mature model of experimental animals for RA. It is also the most commonly used animal model for the screening and study of RA drugs [31]. Thus, in this experiment, we chose the AA model as the research object.

There are many indicators of inflammation associated with RA, such as nitric oxide (NO), nitric oxide synthase (NOS), prostaglandins (PGs), tumor necrosis factor (TNF) and interleukin. NO is a gas, and a sample is not easy to preserve. NO is easy to effuse during storage. Therefore, it is difficult to obtain samples containing NO directly by a microdialysis probe. Microeatotic samples such as TNF-a, PGE_2_, histamine and other inflammatory factors have been reported to evaluate the PD of topical anti-inflammatory drugs [32,33]. PGE_2_, as an important factor for the growth and regulation of cells, is one of the metabolites of arachidonic acid cyclooxygenase, which is commonly found in vivo in tissues. PGE_2_ is one of the important mediators of RA and is thought to play an important role in vascular dilatation, fluid extravasation, and pain in synovial tissue. Increasing evidence shows that PGE_2_ may play a regulatory role in the destruction of articular cartilage [34,35]. In addition, the inhibition of PGE_2_ is considered to be a potential mechanism for treating RA.

It is reported that the determination of PGs in microdialysis samples is mostly carried out by an enzyme-linked immunoassay kit method, but that there is a cross-reaction between different PGs [36]. Research shows that it is feasible to apply a microdialysis sampling technique for collecting PGE_2_ samples [37]. One drawback of limiting PK–PD research is the need for multiple drug tissue concentrations and pharmacological actions at corresponding time points. In this respect, the traditional sampling is not ideal, because the extraction of the sample itself interferes with the PK and PD behavior of the drug. Microdialysis is used to sample the free endogenous and exogenous compounds in the extracellular fluid of the target site in vivo, in real time and online, without interfering with the normal vital signs of the organism. Advantages of this technology include (1) that frequent measurements can be performed, which can provide more information about the shape of the drug concentration–time curve, and allow multiple experiments using the same animal without regard to the blood loss of the small animal; (2) the in vivo determination of the free drug concentration; and (3) that continuous sampling is performed in the case of PK. In this study, the microdialysis technique and UHPLC-MS/MS method were used to detect the contents of PGE_2_ in AA rat articular dialysis fluid. The UHPLC-MS/MS method has a high sensitivity and low detection limit, such that the results showed high reliability and accuracy.

In this study, the concentrations of GE and PGE_2_ were directly measured by UHPLC-MS/MS. GE was significantly increased within 0–2 h. PGE_2_ levels were the lowest at 3 h and increased again at around 4–8 h after the oral administration of different concentrations of GE (30, 60 and 120 mg/kg). The effects of GE on PGE_2_ were observed significantly after reaching the C_max_ of GE (at 2–3 h), suggesting that the inhibitory effect of GE was delayed and that the indirect mechanism mediated the inhibition of PGE_2_. The lag between the drug concentration and PGE_2_ level may have been due to the delay in transportation caused by the distribution of drugs from the circulatory system to the joint cavity. Therefore, an indirect response model was introduced to describe the process.

GE is a kind of iridoid glycoside that is separated from dried and ripened fruits of *Gardenia jasminoide*s Ellis. It has anti-inflammatory, anti-angiogenesis and other pharmacological effects. In recent years, our research group has carried out a large amount of research work on GE’s anti-inflammatory activity and has found that, on the one hand, GE (60 and 120 mg/kg) down-regulated the inflammatory cytokines (IFN-γ, IL-6 and IL-17) secreted by peripheral blood lymphocytes, fibroblast-like synoviocytes and mesenteric lymph node lymphocytes in AA rats, and up-regulated the levels of anti-inflammatory cytokines (IL-4 and TGF-β); on the other hand, GE reduced the degrees of overexpression of Erk1/2, JNK and p38MAPK in peripheral blood lymphocytes, fibroblast-like synoviocytes and mesenteric lymph nodes, and inhibited the MAPKs signal of hyperthyroidism to relieve the AA symptoms [38,39]. From the PK parameter estimates, the apparent volume of distribution is not high, indicating that the GE tissue distribution is not extensive. This finding is consistent with previous reports. On the basis of the UHPLC-ESI-MS/MS method, the bioavailability of GE detected in normal and AA rats in vivo was low, but GE had a high binding with plasma protein. In the organizations, especially inflammation and immune-related tissues were kept in low distributions [40,41]. Although PK–PD modeling has several advantages over classical dose–response studies, no experimental PK–PD studies have been conducted for GE. The study of PK–PD models of anti-inflammatory drugs can provide mechanisms for linking dose and response. Figure 3 shows that PGE_2_ levels in the inflammatory tissue of the model group were significantly higher than those in the normal group, but there was distinct difference between the middle- and high-dose groups of GE (60 and 120 mg/kg) compared with the model group. Compared with the model group, the low dose of GE (30 mg/kg) had no significant differences. The results showed that the middle- and high-dose GE groups could decrease the concentrations of PGE_2_ in the AA rats’ joint cavities. There was no obvious inhibition in the low-dose GE group, which may be because of the low dose or the short time of treatment. This result showed that GE may reduce the concentrations of PGE_2_ in the joint cavities to exert an anti-inflammatory effect to treat AA rats. As can be seen from Figure 4, PGE_2_ did not reach the lowest concentration at the time when GE reached its maximum concentration after 2 h, but did so after 3 h. As a result, there was a lag effect between the maximum concentration of GE and its maximum effect. GE is an effective ingredient of *jasminoidin* that may alleviate the condition by inhibiting the concentrations of cytokine PGE_2_ in patients with RA. Despite this, GE’s deeper mechanism for reducing PGE_2_ still needs further verification.

Since the perfusion liquid does not flow through the probe dialysis membrane for long enough, the dialysis process is in a non-equilibrium state. The drug concentration measured in the dialysate is only a fraction of the actual drug concentration of the extracellular fluid surrounding the probe. In order to obtain the actual concentration of the drug in vivo, the relative recovery of the probe needs to be measured. Factors affecting the relative recovery of the probe are in vitro and in vivo. In vitro factors are physical properties of the probe dialysis membrane (material, pore size and membrane length), the relevant properties of perfusate, the perfusion flow rate, temperature, pH and so on. In vivo factors are the biological rhythm, drug transport, the metabolic rate and the drug and protein binding rate. When the concentrations of GE around the probe are fixed, the recovery rate of the probe decreases with the increase in flow velocity, because the faster the flow velocity, the shorter the balance time and the lower the recovery rate of the drug in and out of the membrane when the flow passes through the dialysis membrane. The lower the perfusion flow rate is, the higher the recovery rate is, which is more conducive to analysis and detection. However, because the samples taken in the binding model study are divided into two parts, they are used for the detection of PK and PD parameters, and the required sample size is relatively large. If the flow rate is too low, this means that the sampling time is longer, which will reduce the time resolution of the microdialysis sampling. On the contrary, when the flow rate increases, the recovery rate will decrease. In the previous in vivo pre-experimental study, the amount of GE entering the joint cavity of the rat was found to be very small, and the in vivo recovery of the joint probe was also low, indicating that the perfusion flow rate was too high. It was troublesome to test the microdialysis sample. Taking into account the narrow area of the joint cavity, the high flow rate was not conducive to the exchange of substances in the body. Considering the recovery rate and the sample volume, the PK experimental conditions in vivo were selected as a flow rate of 1 μL/min and a sampling interval of 60 min.

## 4. Materials and Methods

### 4.1. Materials and Chemicals

GE, a white powder with >98% purity, was purchased from Guangxi Mountain Cloud Biochemical Technology (Beijing, China). The standards for GE (purity of >99.7%) and Pae (purity of >98%) were provided by the National Institute for the Control of Pharmaceutical and Biological Products (Beijing, China). The reference standards for PGE_2_ (purity > 98%), DXM (purity > 98%) and FCA were purchased from Sigma (St. Louis, MO, USA). GTW was purchased from Fuhua Forward Pharmaceutical (Shanghai, China). Heparin sodium injection was obtained from the biochemical pharmaceutical company of Wanbang Biophamaceutical (Hefei, China), and the size was 12,500 units/2 mL. All other chemicals were of analytical grade.

### 4.2. GE and PGE_2_ Assay

Samples were analyzed on an UHPLC-MS/MS instrument (Agilent Technologies, Wilmington, DE, USA) equipped with an Agilent 1290 series liquid chromatography system including a binary pump, an online degasser, an autosampler, a thermostatted column compartment and AB SCIEX Triple Quad 4500 mass spectrometers (AB SCIEX, Framingham, MA, USA) with an electrospray ionization (ESI) source. The software program Analyst 1.6.1 Software was used for the data acquisition and analysis. The column of the ACQUITY UHPLCTM HSS system was an Agilent SB-18 (100 mm × 2.1 mm, 1.7 μm; Waters, Milford, MA, USA), and the flow rate was 0.2 mL/min. The mass spectrometry conditions were set as follows: ESI in negative mode; ion source temperature (TEM) of 400 °C for GE and 550 °C for PGE_2_; ionspray voltage of −4500 V for GE and −4000 V for PGE_2_; curtain gas: 30 psi for GE and 20 psi for PGE_2_; nebulizing gas (Gas 1): 75 psi for GE and 45 psi for PGE_2_; auxiliary gas (Gas 2): 65 psi for GE and 45 psi for PGE_2_. Quantification was performed using MRM in the negative ionization mode at *m*/*z* transitions of 387.4→122.4 for GE and 479.4→449.0 for Pae as an internal standard. Quantification was performed using MRM in the negative ionization mode at *m*/*z* transitions of 351→271 for PGE_2_ and 391→306.7 for DXM as an internal standard. Mobile phase A (acetonitrile) and mobile phase B (0.1% formic acid in water) were perfused in the gradient elution mode (0–1 min, 90% A; 1–4 min, 90%–70% A; 4–7 min, 70%–90% A; 7–9 min, 90% A) with a flow rate of 0.2 mL/min. The injection volume was 2 μL. The mobile phase was composed of methanol (solvent A) and 0.1% formic acid in water (solvent B) for PGE_2_. We made the following gradient program: 0–0.5 min, 30% A; 0.5–3 min, 30%–65% A; 3–5 min, 65%–80% A; 5–7 min, 30% A. The column was kept at a temperature of 25 °C, and the injection volume was 2 μL.

### 4.3. Animals

Specific pathogen-free Sprague Dawley rats weighing 220 ± 20 g were used in this study. All animals were housed under specific pathogen-free conditions with a 12 h light/dark cycle in a temperature-controlled room at 23 °C (±1 °C) and had free access to rat chow and water. All experiments using rats were performed in accordance with protocols approved by the Ethics Review Committee for Animal Experimentation of the Anhui University of Chinese Medicine. Additionally, six rats per group were used for the in vivo microdialysis study.

### 4.4. Experimental Grouping and Administration

The male rats were fed for 1 week for the purpose of adaptation. According to body weight, rats were randomly assigned to six groups, respectively, the blank group; model group; positive drug (GTW) group (40 mg/kg); and the GE low-dose administration group (30 mg/kg), middle-dose group (60 mg/kg), and high-dose group (120 mg/kg). Rats were immunized by a single intradermal injection into the left hind paw with 100 μL of FCA for each rat according to the previous method, and the rats in the normal group received the same volume of saline.

### 4.5. Microdialysis Sampling

Joint microdialysis systems consisted of a microdialysis syringe pump (CMA400, Stockholm, Sweden), microsyringe (2.5 mL; CMA, Stockholm, Sweden), and refrigerated fraction collector (CMA470, Stockholm, Sweden). The dialysis probes for the joints (4 mm in length) were U-shaped and made of a hollow cellulose fiber (membrane diameter: 0.5 mm in inner diameter; CMA12, Stockholm, Sweden). These were used for both the in vitro and in vivo studies and had a molecular weight cutoff of 20,000 Da. The perfusate was filtered with 0.22 μm membranes.

After successful modeling, the rats were given intragastric administration. Rats were subjected to surgery on the seventh day of continuous gavage. An intraperitoneal injection of 20% urethane (0.6 mL/100 g) was used to anesthetize the rats.

A Ringer’s solution consisting of 147 mM sodium chloride, 2.2 mM calcium chloride, and 4 mM potassium chloride containing GE (0.01 mg/mL) was perfused through the probe at a rate of 1 μL/min. After a 1 h stabilization period following the implantation of the microdialysis probe, the rat was administered with GE. The dialysis samples were collected every 30 min for 6 h as PD basic values and drug control. Then, oral administration of medicine to rats was performed in accordance with the “4.4” after 1 h of stabilization. The microdialysis samples were collected every 60 min for 8 h. The samples were divided into two parts. One was measured to determine the contents of GE, and the other was used for the determination of PGE_2_ contents; only PGE_2_ was measured in the positive group.

### 4.6. Study on *in Vitro* and *in Vivo* Recovery of Microdialysis Probe

For PK research, concentration and velocity are the two main variables. It is very important to ensure that the recovery rate of probes is independent of the concentration explored in the whole PK experiment, because the concentration of the analyte in the joint cavity is fluctuant. The recovery rate and release rate of the probes were determined by incremental and decremental methods through in vitro recovery experiments.

The dialysis membrane of the probe was completely immersed in a Ringer’s solution containing 100 μg/mL of GE; a blank Ringer’s solution was used as a perfusion solution, and the probes were perfused at different flow rates (0.5, 0.8, 1 and 2 μL/min). Four samples of the dialysate were collected at each flow rate. The concentrations of the drug (C_dialysis_) in the dialysate and the concentration of the drug in the perfusate (C_perfusate_) were determined by UHPLC-MS/MS. The relative recovery of the GE probe was calculated by the incremental method. The formula is as follows:

RR = C_dialysis_/C_perfusate_ × 100%. (1)

The reduction method was as follows: The dialysis membrane of the probe was completely immersed in a blank Ringer’s solution, and the probe was perfused with GE solution at a concentration of 100 μg/mL as a perfusate at different flow rates (0.5, 0.8, 1 and 2 μL/min). The four samples of the dialysate were collected at each flow rate, and samples were collected.

RL = (C_perfusate_ − C_dialysis_)/C_perfusate_ × 100%. (2)

The effect of concentrations on the recovery rate was investigated as follows: The microdialysis probe was perfused with Ringer’s solution containing GE at low, medium, and high concentrations (50, 100 and 200 μg/mL) at a flow rate of 1 μL/min. Four dialysate samples were collected at each concentration. The in vivo recovery of GE was calculated using the following equation:

Relative recovery rate (RR) = C_dialysis_/C_perfusate_ × 100%. (3)

The microdialysis probe was completely immersed in a 50 mL beaker of blank Ringer’s solution and was perfused with three different concentrations of GE (50, 100 and 200 μg/mL) at a rate of 1 μL/min. Four samples of the dialysate were collected at each concentration. The loss rate of the GE joint probe was calculated by the reduction method.

RL = (C_perfusate_ − C_dialysis_)/C_perfusate_ × 100%. (4)

The anti-dialysis method was used to investigate the in vivo recovery of microdialysis probes. The probe was perfused at a rate of 1 μL/min with Ringer’s solution containing 100 μg/mL of GE. The dialysis samples were collected every hour to investigate the stability of the microdialysis probe over 8 h.

R _in vivo_ =(C_perfusate_ − C_dialysis_)/C_perfusate_ × 100%. (5)

### 4.7. GE PK Parameters

The PK parameters included C_max_ for the maximum concentration, T_max_ for the time to reach C_max_, t_1/2_ for the elimination half-life, AUC for the area under the curve, V/F for the apparent volume of distribution, and CL/F for the apparent clearance rate of GE; they were calculated by a non-compartment model using Phoenix 6.4 (Princeton, NJ, USA). The peak concentration (C_max_) and peak plasma time (T_max_) were directly obtained. The area under the curve (AUC) of the function relating GE levels to time was calculated by using the trapezoidal rule. The parameters’ clearance (CL/F) and volume of distribution (V/F) were calculated by standard methods [42], where Vd = D/C_max_ (C_max_ is the maximal plasma concentration) and CL = Ke × Vd (Ke is the rate constant of elimination).

### 4.8. PK–PD Modeling

The integrated PK–PD model was developed to establish the time–concentration–response relationship of GE. In the GE PK–PD relationship study, the joint cavities’ dialysate concentrations of GE and PGE_2_ were used. As a time delay between the concentrations and their effects was observed, a PK–PD model with a separated effect compartment was used for the analysis of the data. PK was described by a linear one-compartment model with first-order elimination. The equations that described the GE concentrations are as follows:

*C(T)* = *D* × *K*01/*V*(*K*01 − *K*10) × (*EXP*(−*K*10 × *T*) − *EXP*(−*K*01 × *T*)) (6)

*K*10 = *CL*/*V* (7)

where *D* is the dose, *V* is the volume of distribution and *T* is the time.

On the basis of the new mechanism of GE on PGE_2_, an indirect response model and the following equation were used to describe the inhibitory effect of GE on the concentrations of PGE_2_. A dynamic model of PGE_2_ concentrations was established.

*dR*/*dT* = *Kin*(1 − (*Cp*/*Cp* + IC_50_)) − *Kout* × *R* (8)

where *K_in_* and *K_out_* are system-dependent parameters, the production of the response and the first-order rate constant for the dissipation of the response, respectively. R is the observed response parameter. IC_50_ is the concentration that resulted in 50% of the maximum stimulation. *Cp* is the concentration of GE.

The following parameters of the PK–PD model were evaluated: IC_50_, *K_in_* and *K_out_.*

### 4.9. Statistical Analysis

PK and PK–PD parameters were found using Phoenix 6.4 software (Princeton, NJ, USA). The data are represented as the means ± SD. Comparisons among more than two groups were performed using one-way analysis of variance followed by Dunnett’s test. Comparisons between two groups were performed using the unpaired Student’s *t*-test. The statistical significance was set at *p* < 0.05.

## 5. Conclusions

On the basis of the determination of GE concentrations and PGE_2_ efficacy levels in the dialysate, the developed methods were successfully applied to study PK–PD modeling of GE’s effect on decreasing PGE_2_ release after the oral administration of GE in AA rats. The established PK–PD model characterized the dose–effect relationship of GE and could be used to predict the effect of GE on decreasing PGE_2_ release in AA rats; it might also provide reference for further pre-clinical and clinical PK–PD studies of GE. When selecting a suitable endogenous micromolecule as an effect marker, microdialysis in AA rats is an appealing technology for PK–PD studies.

## Figures and Tables

**Table 1:** Effects of perfusion flow rate on the recovery by gain and by loss from the knee-joint microdialysis probes in vitro (*n* = 4, $\bar{x}$ ± *s*).

Current Speed (μL/min)	0.5	0.8	1	2
Incremental method Realtive recovery (RR) (%)	56.45 ± 3.44	52.87 ± 4.07	48.05 ± 5.41	25.63 ± 4.30
Decremental method Relative loss (RL) (%)	53.49 ± 2.75	50.66 ± 5.00	44.42 ± 4.46	23.87 ± 3.82

**Table 2:** Effects of GE concentrations on the in vitro recovery by gain and by loss from the knee-joint microdialysis probes in vitro (*n* = 4, $\bar{x}$ ± *s*).

Concentration (μg/mL)	50	100	200
Incremental method RR (%)	27.09 ± 0.78	28.98 ± 0.80	28.07 ± 0.37
Decremental method RL (%)	26.91 ± 1.01	30.41 ± 0.30	29.91 ± 0.66

**Table 3:** Non-compartmental PK parameters of GE microdialysate levels obtained from articular cavities of AA rats: AUC (area under the curve), Cl/F (clearance of distribution), V/F (steady state volume of distribution), C_max_ (extrapolated maximal concentration), T_max_ (peak time) and t_1/2_ (elimination half-life) after oral administration of GE (30, 60 and 120 mg/kg).

PK Parameters	Oral Administration Dose (mg/kg)		
	Low (30)	Medium (60)	High (120)
t_1/2_	0.636 ± 0.057	0.726 ± 0.132	0.665 ± 0.042
T_max_ (h)	2	2	2
C_max_ (ng/mL)	124.479 ± 2.147	469.258 ± 37.999	805.906 ± 15.81
AUC_0-t_ (ng/mL×h)	283.434 ± 20.924	1477.928 ± 80.7	2074.373 ± 113.885
AUC_0-inf_ (ng/mL×h)	284.624 ± 20.052	1483.883 ± 81.050	2081.072 ± 134.014
V/F (L/kg)	96.460 ± 7.512	42.364 ± 7.595	55.461 ± 4.756
CL/F (L/h/kg)	105.803 ± 6.839	40.529 ± 2.081	57.867 ± 3.811

Data are reported as means (coefficient of variation, %) of six animals.

**Table 4:** PK–PD parameters estimation for the treatment effect of GE by the application of the E_max_ model in AA rats.

	Variable								
	IC_50_			*K_in_*			*K_out_*		
Dose (mg/kg)	Mean	SD	SE	Mean	SD	SE	Mean	SD	SE
30	0.74 *	0.23	0.1	523.75 *	390.79	159.54	0.97	0.72	0.29
60	2.62	0.44	0.18	556.5 *	170.94	69.78	1.08	0.32	0.13
120	1.81	0.4	0.17	272.78	103.67	42.32	0.53	0.2	0.08

*K_in_* and *K_out_* are system-dependent parameters that adequately account for the zero-order input rate constant for the production of response and first-order rate constant for the dissipation of response, respectively. IC_50_ is the concentration that resulted in 50% of the maximum stimulation. Data are reported as means (coefficient of variation, %) of six animals; * *p* < 0.05 vs. GE (120 mg/kg) group.
